# Microsecretory adenocarcinoma: simplifying the diagnosis of a recently recognized salivary gland and cutaneous adnexal neoplasm

**DOI:** 10.1186/s13000-025-01628-z

**Published:** 2025-04-02

**Authors:** Nancy A. Abdelmoneim, Aya M. Elfouly, Nourhan A. Abou Madawi

**Affiliations:** Oral Pathology Department, Faculty of Dentistry, Alexandria University, Champollion Street, Azarita, Alexandria 21521 Egypt

**Keywords:** Microsecretory adenocarcinoma, Adenocarcinoma not otherwise specified, Salivary gland tumors, Immunohistochemistry, FISH, RNA sequencing

## Abstract

**Background:**

Microsecretory adenocarcinoma (MSA) is a newly identified entity in the WHO classification of salivary gland tumors characterized by *MEF2C::SS18* fusion. It was previously considered as adenocarcinoma not otherwise specified (NOS). With the discovery of new gene fusions specifying distinct salivary gland tumors and restricting the diagnosis of adenocarcinoma NOS, five cases of MSA were recognized for the first time using targeted RNA sequencing. Afterwards, further authors reported MSA in the salivary glands and more recently in the skin.

**Methods:**

We reviewed the literature for all cases of MSA reported in English-language articles. We comprehensively discussed clinical, histopathological, immunohistochemical and molecular findings of the retrieved cases.

**Results:**

Forty cases were identified. Thirty cases occurred in the salivary glands and ten cases occurred in the skin. They were characterized histologically by a well circumscribed mass formed of microcysts containing basophilic secretions and enclosed in a fibromyxoid stroma. The tumor cells were flattened resembling intercalated duct cells with minimal eosinophilic cytoplasm and small oval nuclei. By immunohistochemistry, the tumor cells were positive for SOX10, S100, p63 and negative for p40, calponin and mammaglobin. However, cutaneous cases had a somewhat different immunoprofile.

**Conclusion:**

MSA is a salivary gland malignancy that also has a cutaneous counterpart. Focusing on emphasising the almost consistent histopathological and immunohistochemical findings help in increasing the awareness of clinicians, surgeons and pathologists about it and at the same time lessening the need for more complicated diagnostic methods that are not readily available in all institutions. Despite the low-grade nature of this tumor, thorough management and rigorous follow up of cases are highly recommended due to occasional aggressive behaviour.

## Introduction

Salivary gland tumors (SGTs) are characterized by a great diversity in morphology and genetic profile in comparison with tumors of other organs [1, 2]. The overlap of histologic and immunohistochemical features between various salivary gland neoplasms is a main cause of diagnostic challenge. Tumors that fail to meet common well-recognized characteristics are diagnosed as adenocarcinoma not otherwise classified (NOS). The term adenocarcinoma NOS is given to a salivary gland carcinoma that forms ducts and/or glandular structures but does not fit into a more distinct category. It is, thus, a diagnosis of exclusion [3]. Here comes the importance of molecular analysis as a gold standard in redefining and identifying new tumors that were previously misclassified [4]. Targeted RNA sequencing (RNA-Seq) is a molecular diagnostic assay that can detect novel gene fusions [5]. The discovery of gene fusions that characterize certain groups of SGTs has dramatically changed the classification of these tumors in the last decade; where novel tumor entities have been recognized and added according to their distinctive molecular profile [6].

Microsecretory adenocarcinoma (MSA) is an example of a newly identified entity in the WHO classification of SGTs that was previously misclassified as adenocarcinoma NOS [1]. It is a low-grade malignancy first described by Bishop et al. in 2019 [7], characterized by a unique *MEF2C::SS18* fusion resulting from chromosomal translocation between gene *MEF2C* (5q14.3) and gene *SS18* (18q11.2). In addition to salivary glands, MSA can also occur on the skin and represents an analogue of salivary MSA [8, 9].

Cutaneous adnexal tumors, like SGTs, comprise a group of lesions that cannot be readily subclassified into a known entity and are also called Not Otherwise Specified [9]. Following the characterization of salivary gland MSA, pathologists started encountering cutaneous lesions with a similar histological and molecular profile. These cutaneous MSAs usually present as nodules at acral locations with an indolent clinical course [9].

Molecular analysis has undoubtedly minimized the spectrum of SGTs diagnosed as adenocarcinoma NOS by recognizing unique entities. However, it is crucial to understand its limitations, for instance, negative results of molecular tests cannot fully exclude certain diagnosis due to multiple genetic abnormalities existing within tumors [10]. Moreover, molecular tests, especially next-generation sequencing (NGS) are expensive and are not readily available in all laboratories especially in developing countries. NGS also requires a significant amount of RNA that is not always available from archived cases or small biopsies [11]. Thus, hopefully with the growing recognition of MSA, enhancing pathologists’ understanding of its histological features would deem molecular testing unnecessary in clear-cut cases. In such instances, the diagnosis can be reliably confirmed through immunophenotypic analysis.

This review aims to summarize important points concerning MSA in terms of clinical, histological, immunohistochemical, and genetic characteristics to enlighten surgeons and pathologists about this new neoplasm. We also included photomicrographs of a straightforward microsecretory adenocarcinoma case diagnosed at our Oral Pathology department to further strengthen the assumption of excluding molecular tests for a simpler definitive diagnosis.

## Review and discussion

We conducted a search in Web of Science, PubMed, Google Scholar and Scopus databases using the keyword “Microsecretory adenocarcinoma” for English written articles. A total of 40 reported cases were identified. They involved 30 cases of MSA arising in the salivary glands of which six cases were reported twice [6, 7, 12] and ten cases arising in the skin.

### Clinical features

The majority of MSA cases occurred in the minor salivary glands: most commonly the palate followed by the buccal mucosa (Table 1). Two cases were reported in the parotid gland [6, 13]. MSA can also develop in the skin, in different parts including external auditory canal, nose, chin, scalp, hand and eyelid. MSA presents as a slowly growing painless mass with size ranging from 0.6 to 5.3 cm (mean, 1.3 cm). There is a wide age range; between 17 and 89 years and there is a slight female predilection (Table 1).

**Table 1:** Clinical features of microsecretory adenocarcinoma

Reference	Number of cases	Original diagnosis	Age in years	Sex	Tumor site	Tumor size in cm	Follow up in months
Salivary cases							
Bishop et al., 2019 [7]	5	Adenocarcinoma NOS (3) Polymorphous adenocarcinoma (2)	21–80	M = 2 F = 3	Palate (2) Buccal mucosa (2) Parotid gland (1)	0.8–2.2	N/A
Kawakami et al., 2020 [12]	1	Microsecretory adenocarcinoma	37	F	Palate	1.5	4 No recurrence
Walsh et al., 2021 [14]	1	Adenocarcinoma NOS	35	F	Palate	1.5	N/A
Bishop et al., 2021 [6]	24 (18 new cases and six previously reported cases)	Adenocarcinoma NOS (11) Microsecretory adenocarcinoma (7) Polymorphous adenocarcinoma (4) Adenoid cystic carcinoma (1) Secretory myoepithelial carcinoma (1)	17–83	M = 11 F = 13	Palate (14) Buccal mucosa (6) retromolar trigon (2) Angle of mandible (1) Parotid gland (1)	0.6-3	1-216 months No recurrence
Gui et al., 2022 [15]	1	Adenocarcinoma presented with lymph node metastasis	70	M	Palate	5.3	20 No recurrence
Hosseini et al., 2022 [16]	1	Microsecretory adenocarcinoma	43	F	Palate	0.8	12 No recurrence
Jurmeister et al., 2022 [17]	1	Low grade Mucoepidermoid carcinoma Pleomorphic adenoma	62	M	Buccal mucosa	N/A	84 Recurrence and lung metastasis
Lu et al., 2024 [4]	1	Microsecretory adenocarcinoma	57	F	Palate	1.4	20 No recurrence
Razack et al., 2024 [13]	1	Microsecretory adenocarcinoma	48	M	Parotid gland	3.5	N/A
**Cutaneous cases**							
Chan et al., 2022 [18]	1	Chondroid syringoma	44	F	External auditory canal	1.9	1.5 No recurrence
Dibbern et al., 2023 [19]	1	Ceruminous carcinoma	89	F	External auditory canal	N/A	N/A
Bishop et al., 2023 [8]	4	Microsecretory adenocarcinoma	61–74	M = 4	Nose Chin Posterior scalp External auditory canal	N/A	3–6 No recurrence
Bogiatzi et al., 2023 [9]	3	Syringoma Adenoid cystic carcinoma Sweat gland carcinoma NOS Cribriform carcinoma Secretory carcinoma	53–58	M = 1 F = 2	Forearm Fourth finger of the hand Frontoparietal area	0.9–1.3	17–45 No recurrence
Novice et al., 2024 [20]	1	Microsecretory adenocarcinoma	58	M	Eyelid	0.7	N/A

*N/A* not available

M male

F female

Six cases in [6] were reported previously in [7] and [12]

### Histopathological features

**Fig. 1:**
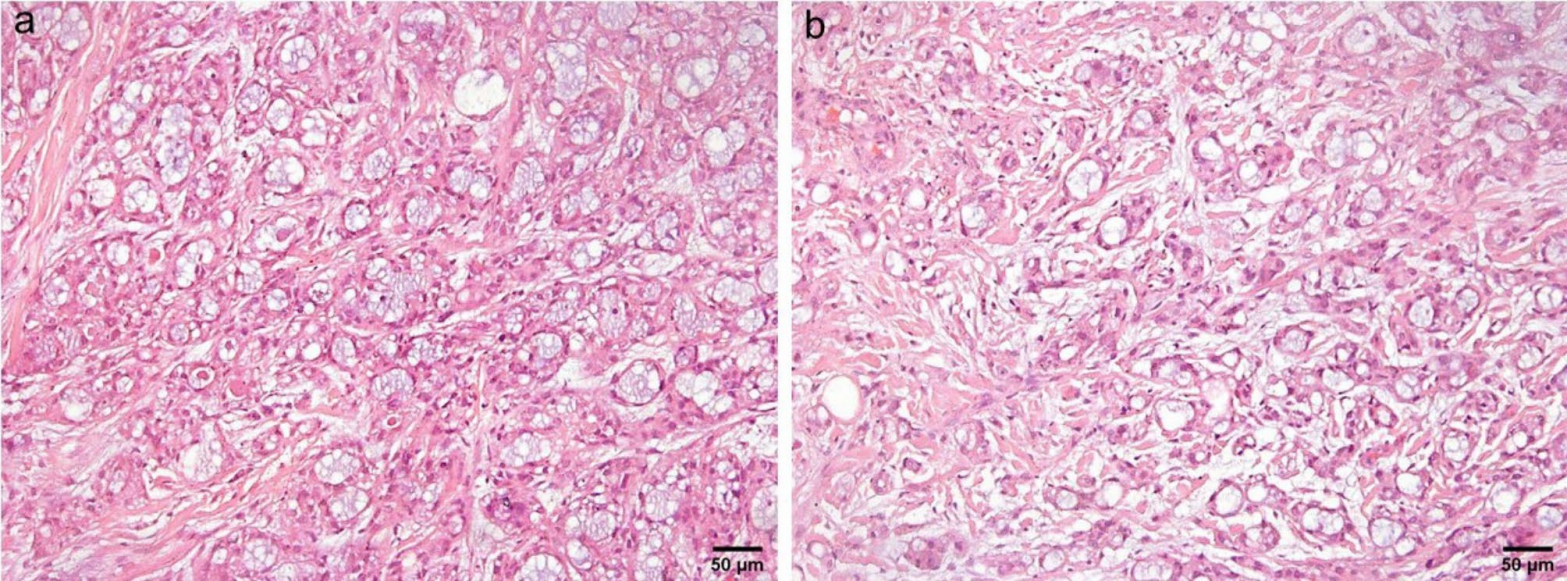
Microsecretory adenocarcinoma is composed of microcysts and tubules containing basophilic secretions and enclosed within a fibromyxoid stroma (**a** &**b**) (H&E, x200)

**Fig. 2:**
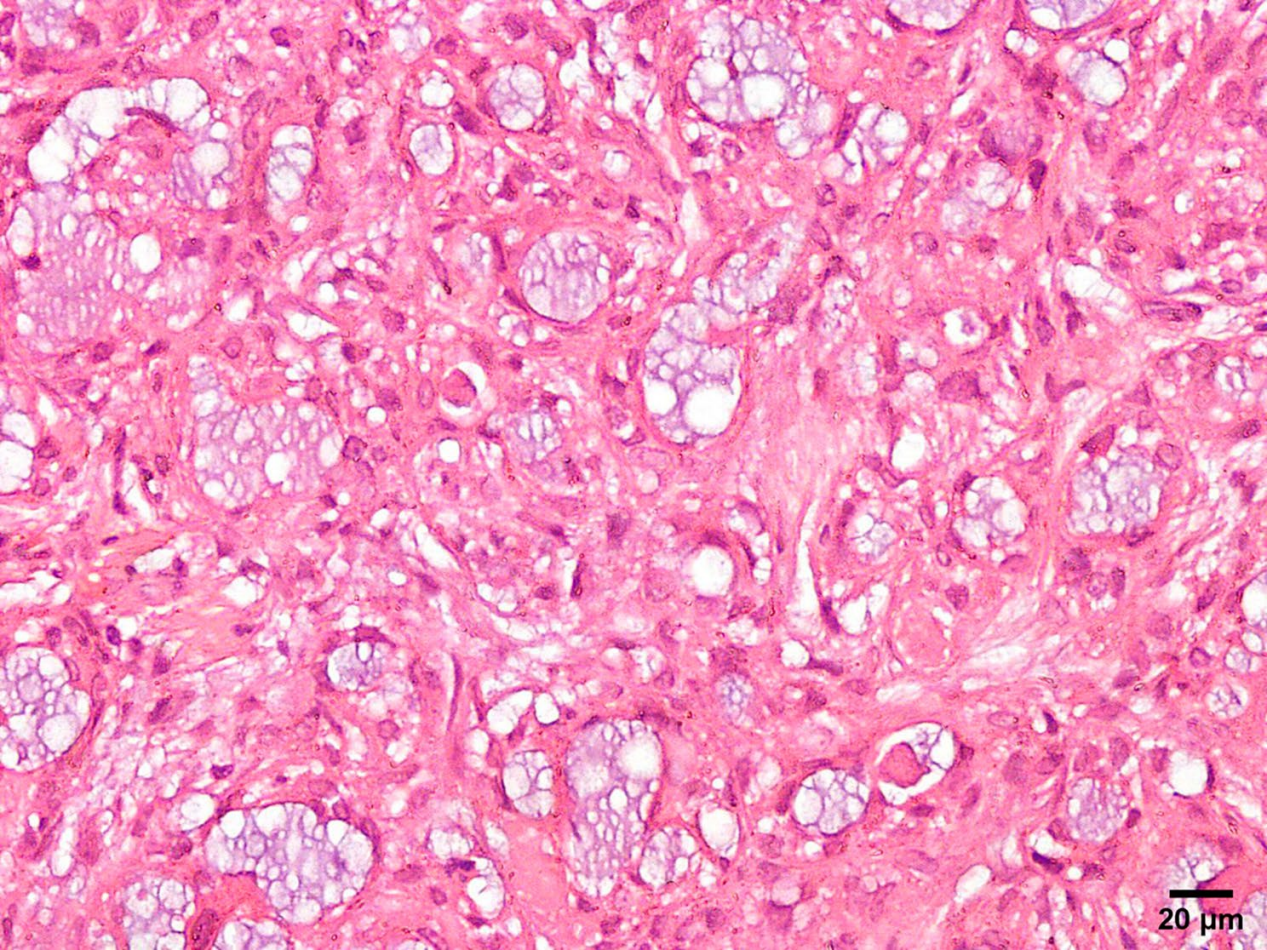
The microcysts and tubules are lined by flattened cells with minimal eosinophilic cytoplasm and small oval nuclei (H&E, x400)

The reported cases of salivary and cutaneous MSA share basic characteristic histological features that are more or less consistent among the majority of cases, although few cases exhibited variations. Low magnified microscopic view reveals a well circumscribed unencapsulated mass. The tumor cells proliferate in the form of microcysts, tubules, trabeculae and cords. A cribriform pattern is uncommon, seen in only two of the reported salivary gland cases [7] and three of the cutaneous cases [9]. Abundant basophilic luminal secretions and a fibromyxoid stroma of variable cellularity are characteristic (Fig. 1). Fibrohyaline stroma is not common, found in only three cases. Mitotic rate is low, around 0–1 mitotic figures in every ten high-power fields [7]. The tumor cells resemble intercalated ductal cells with mild eosinophilic to clear cytoplasm, uniform oval hyperchromatic nuclei and inconspicuous nucleoli [1]. The cells mostly have a flattened appearance [21] (Fig 2). Occasional findings include pseudoepitheliomatous hyperplasia; encountered in six salivary gland cases [6, 16] and three cutaneous cases [9]. Another finding is tumor-associated lymphoid proliferation cuffing the tumor; found in four salivary gland cases [6] and three cutaneous cases [9]. Metaplastic bone formation and psammomatoid calcifications are uncommon findings found in two and one salivary gland cases respectively [6]. One case showed solid growth with a spindle cell component, rosette like structures and clear cells, besides the standard histology of MSA in the series reported by Bishop et al. [6]. Spindle cell component was also detected in a case of cutaneous MSA as reported by Walsh et al. [14]. Three salivary gland cases revealed perineural invasion [4, 6, 15]. Lymphovascular invasion on the other hand appeared in only one salivary gland case with high grade transformation that also revealed necrosis, high mitotic rate (10/10 high-power fields) and metastasis to lymph nodes [15]. However, there was also a cutaneous case exhibiting high mitotic rate, nuclear pleomorphism, open chromatin and necrosis [8]. This means that two out of the 40 reviewed cases had focal areas of high-grade transformation [8, 15].

**Fig. 3:**
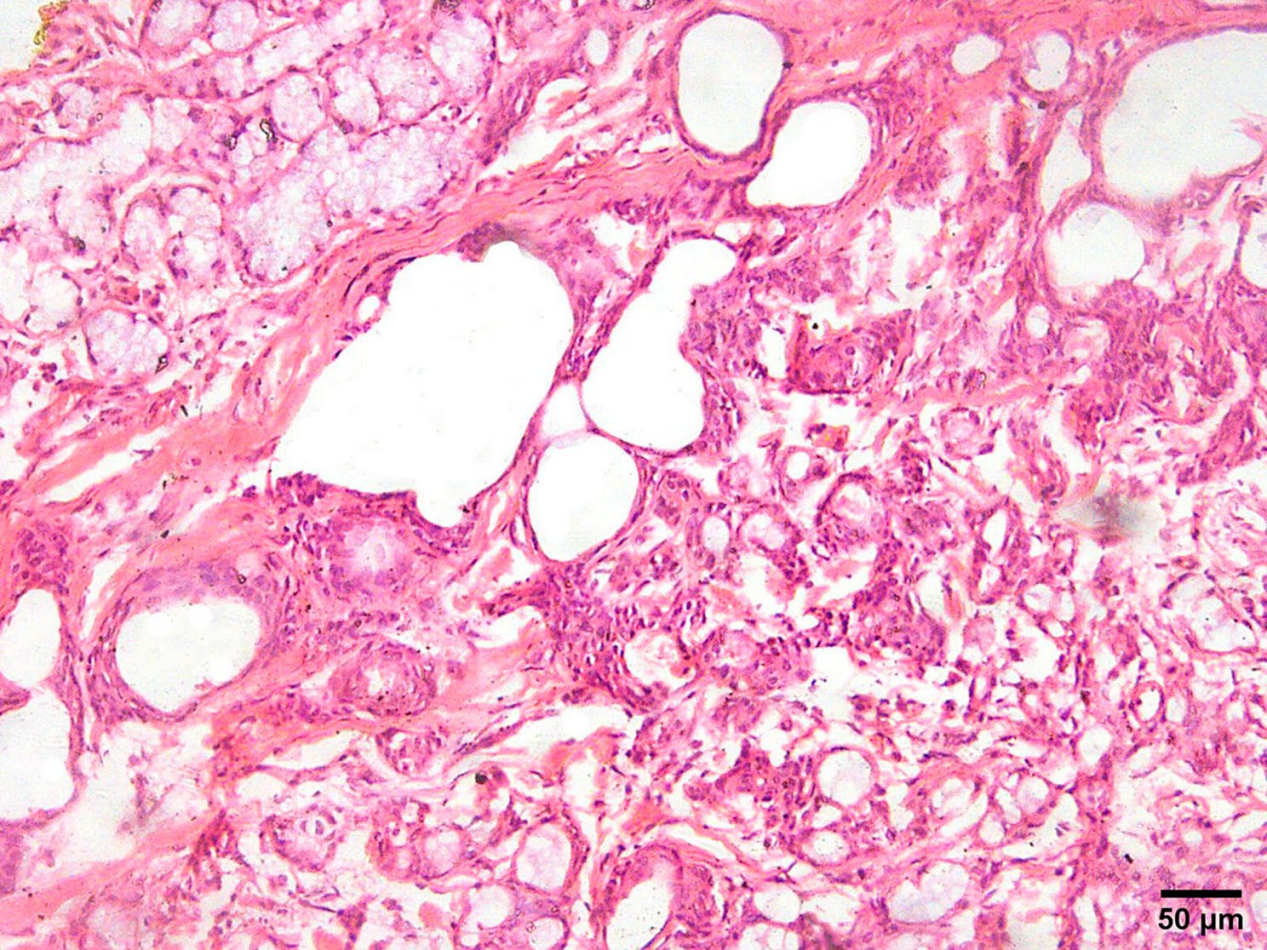
Infiltration of tumor nests into adjacent normal salivary gland tissue (H&E, x200)

Subtle infiltration into nearby tissues is noted upon higher magnification of microscopic examination (Fig. 3). Despite the tumor’s bland cytologic appearance and lack of metastasis in the majority of cases, the destructive invasion of surrounding structures justifies its classification as a carcinoma [21].

### Immunohistochemical features

**Fig. 4:**
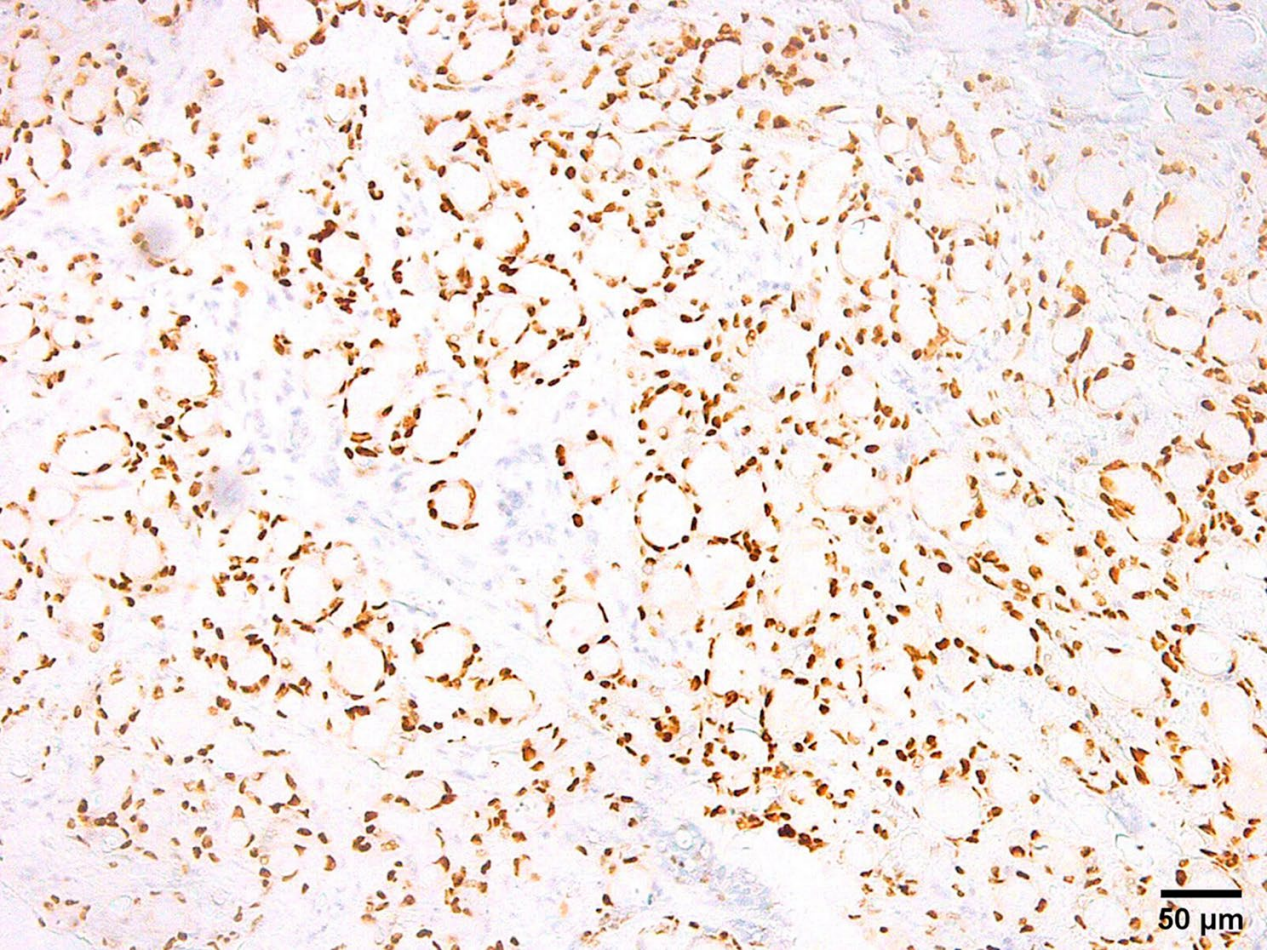
Microsecretory adenocarcinoma is positive for SOX 10 (SOX10 immunostain, x200)

**Fig. 5:**
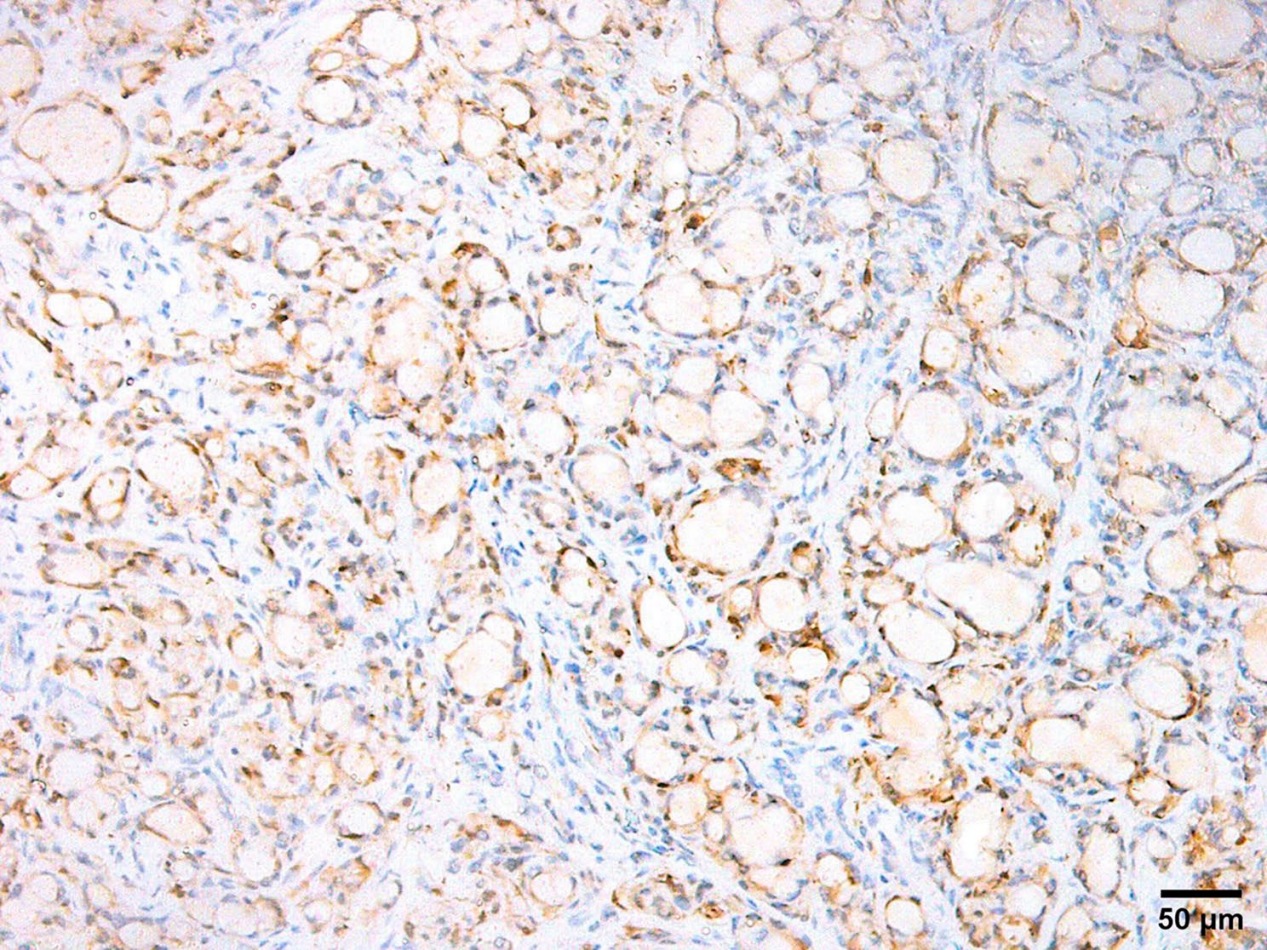
Microsecretory adenocarcinoma is positive for S100 (S100 immunostain, x200)

**Fig. 6:**
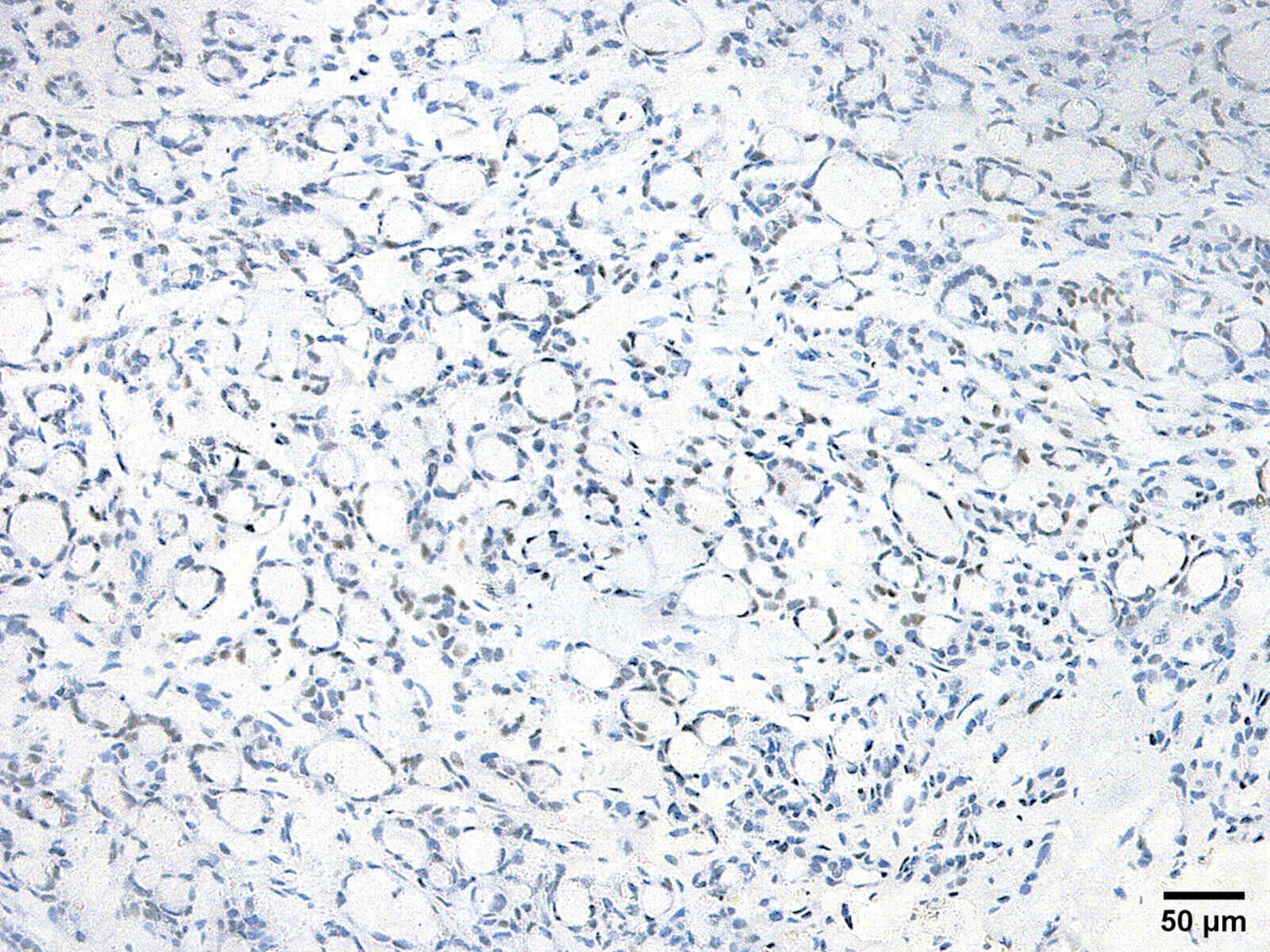
Microsecretory adenocarcinoma is positive for p63 (p63 immunostain, x200)

Salivary MSA has a distinctive immunoprofile that delineates it from other salivary gland entities. A discrepant immunophenotype of p63 positivity along with a negative reaction to its isoform p40 characterizes this tumor. The reaction to p63 can be focal in some cases. Tumor cells often demonstrate positivity for S100 and SOX10 while they are consistently negative for calponin and mammaglobin. Smooth muscle actin is focally positive in some cases (Table 2) (Figs. 4, 5 and 6).

**Table 2:** Immunohistochemical features of microsecretory adenocarcinoma

Reference	Number of cases	S-100	SOX10	p63	p40	Calponin	Mammaglobin	Smooth muscle actin
Salivary cases								
Bishop et al., 2019 [7]	5	5/5	N/A	5/5	0/5	0/5	0/5	0/5
Kawakami et al., 2020 [12]	1	+	+	+	-	-	N/A	-
Walsh et al., 2021 [14]	1	+	N/A	+	-	-	N/A	N/A
Bishop et al. 2021 [6]	24	24/24 Weak positivity in two cases Focal positivity in one case	14/14	24/24 Focal positivity in five cases	0/21	0/12	0/16	4/20 Focal positivity
Gui et al., 2022 [15]	1	+	+	+	-	-	-	-
Hosseini et al., 2022 [16]	1	+	+	+	-	N/A	-	N/A
Jurmeister et al., 2022 [17]	1	N/A	+	+	N/A	N/A	N/A	N/A
Lu et al.,2024 [4]	1	+	+	+	-	N/A	-	Focally +
Razack et al., 2024 [13]	1	+	+	+	-	-	-	N/A
**Cutaneous cases**								
Chan et al., 2022 [18]	1	+	+	+	-	N/A	N/A	N/A
Dibbern et al., 2023 [19]	1	+	N/A	+	-	N/A	-	N/A
Bishop et al., 2023 [8]	4	4/4	N/A	4/4	2/4	2/4	N/A	2/4
Bogiatzi et al., 2023 [9]	3	2/2 Focal positivity	3/3 Diffuse positivity	3/3 Partial positivity	1/2 Weak positivity	N/A	1/3	2/3
Novice et al., 2024 [20]	1	+	N/A	-	N/A	N/A	N/A	N/A

/ numerator is the number of positive cases, denominator is the total number of cases in which the assay was done

N/A not available

Six cases in [6] were reported previously in [7] and [12]

Ki-67 proliferative index can be a good indicator of MSA grade as reported by Gui et al. [15]. Standard MSA has low proliferative index but with progression into high grade morphological status, the proliferative index markedly increases. Furthermore, positive p63 expression helps in confirming the diagnosis of MSA and determining its grade. Loss of p63 implies the aggressive nature of the tumor [15].

Ten cases of cutaneous MSA are reported so far. They have positive immune reaction to SOX10, S100 and p63. However, focal or partial positivity for S100 and p63 were noted in some cases [9, 18]. One case didn’t express p63 [20]. As regards p40, some cases showed positive expression in abluminal cells lining tubules, these same cells also stained positively for smooth muscle actin or calponin, suggesting a true myoepithelial cell origin [8]. Mammaglobin was focally positive in only one case [9] (Table 2).

### Differential diagnosis

Pathologists encounter difficulty in diagnosis of SGTs because they are rare tumors, characterized by a great morphologic diversity with overlap of histological features between different types. Also, immunohistochemistry (IHC), despite being an important aid, does not provide a unique picture for each tumor. Moreover, there are technical challenges such as the limited availability of tissue for auxiliary studies [17].

Histological examination of biopsies is a crucial and indispensable step in deciding the exact diagnosis. It should be done with precision and rigor. Initially, examining slides at low power gives a primitive indication of certain SGTs from the predominant color of the H & E-stained slide. For example, adenoid cystic carcinoma (AdCC) and polymorphous adenocarcinoma (PAC) appear more basophilic while secretory carcinoma (SC) and mucoepidermoid carcinoma (MEC) appear more eosinophilic [10]. Next, determining the types of cells that compose the tumor and the nature of extracellular matrix guide the pathologists to specific tumors. Additionally, some findings assert malignancy such as nerve invasion, lymphovascular invasion, margin infiltration and extensive collagenization of stroma [17].

IHC is important in assessing the composition of the tumor and the nature of its cells [22]. It is the second aid after hematoxylin and eosin (H & E) -based histology to help confirming diagnosis [23]. p63, a homologue of the p53 tumor suppressor gene, is normally expressed in the basal layer of stratified epithelium, myoepithelial cells, and epithelial neoplasms. In SGTs, it is expressed in abluminal cells (basal and myoepithelial cells) and cells of squamous differentiation. AdCC, epithelial myoepithelial carcinoma, pleomorphic adenoma, PAC, myoepithelioma and MEC show positivity to p63. p63 antibody cannot differentiate between true and pseudo-myoepithelial lineages [22]. SOX10 and S100 are also myoepithelial markers, but, like p63, they are not specific to myoepithelial cells. p40, an isotype of p63, is more specific than p63. Tumors of myoepithelial origin tend to display a congruent positive p63/p40 staining scheme. Lack of p40 expression negates the myoepithelial cell nature [22].

MSA exhibits intercalated duct-like tumor cells that do not express p40 [15, 22]. The p63+/p40- immunophenotype is suggestive of a progenitor cell state like the intercalated duct cells [15]. The focal smooth muscle actin reactivity encountered in some reported cases may be due to the tendency of intercalated duct-like cells to display such a focal expression as in PAC [6]. Furthermore, the possibility of presence of actual neoplastic myoepithelial cells in some tumors was recently described in salivary intercalated duct-type intraductal carcinoma [24]. Being aware of MSA’s unique immune profile could help in differentiating cases with a cribriform pattern from AdCC.

It is also important to be aware of other newly diagnosed entities and for pathologists to be enlightened about them. Two entities of SGTs have been discovered and categorized before MSA; secretory myoepithelial carcinoma (SMC) and sclerosing microcystic adenocarcinoma (SMA), in 2013 [25] and 2016 [26] respectively. Like MSA, they also display a low -grade morphology along with secretory material [27].

SMA closely resembles cutaneous microcystic adnexal carcinoma. In SMA, tumor cells grow in the form of infiltrative tubules, cords and nests in a characteristic voluminous densely collagenized stroma. It has a characteristic biphasic morphology, with peripheral myoepithelial cells (positive for S100, smooth muscle actin, p63 and p40) and luminal cuboidal ductal cells (positive for CK7 and pan cytokeratin). Ductal structures contain eosinophilic secretory material positive for mucicarmine. Until now, SMA appears not to have a characteristic molecular profile [26, 27]. The concordant p40 and p63 positivity, along with the lesser cellularity and more abundant desmoplastic stroma helps differentiate SMA from MSA. Moreover, SMA commonly shows perineural invasion which is rare in MSA [1].

SMC has been previously named mucinous myoepithelioma [25], but its low-grade infiltrative pattern along with its ability to give both mucinous and serous secretions make the later designation more accurate. SMC demonstrates characteristic signet ring cells with intracytoplasmic vacuoles containing mucous or eosinophilic serous secretions. These intracytoplasmic vacuoles help differentiate it from MSA. The unique SS18 rearrangement can also help distinguish MSA from SMC [27].

As for the histologic difference between MSA and SC, SC cells have abundant, often vacuolated eosinophilic cytoplasm and prominent nucleoli while MSA cells are sometimes plump but mainly flat with scanty cytoplasm [21]. Although both tumors are positive for S100 and SOX10 and negative for p40, they differ in their reactivity to other immune markers. SC is positive for mammaglobin and negative for p63 in contrast to MSA [1].

Mucinous adenocarcinoma is a rare SGT that bears a histologic resemblance to MSA due to the abundant mucin secretion in both. However, mucinous adenocarcinoma has a macrocystic and papillary architecture with sheets of non-cohesive signet ring cells, unlike the uniform microcystic configuration detected in MSA. Also, mucinous adenocarcinoma is negative for SOX10, S100 and p63 [6, 27, 28].

**Fig. 7:**
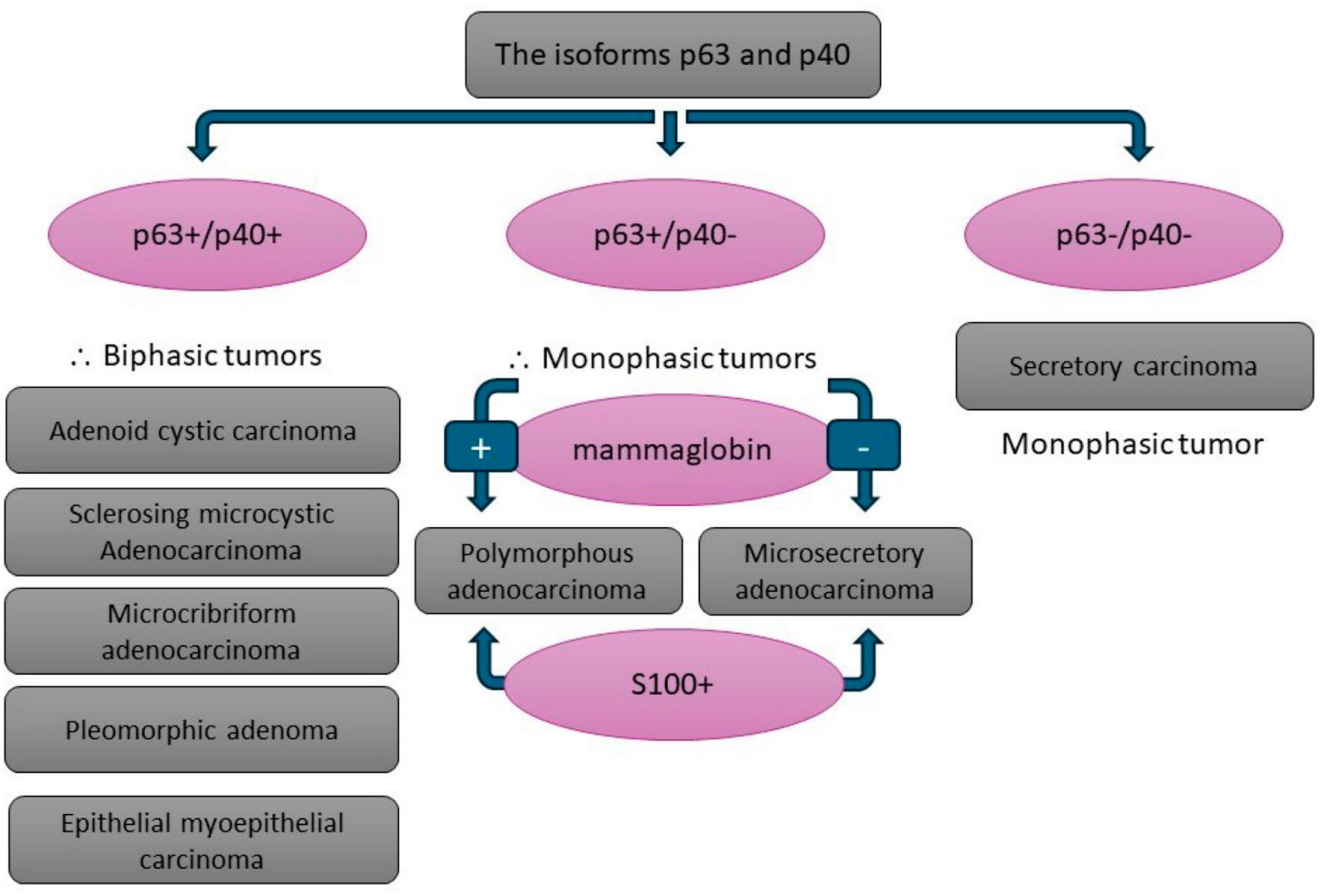
Schematic diagram simplifying the differential diagnosis of Microsecretory adenocarcinoma immunohistochemically

PAC exhibits an intercalated duct-like morphology and possible microcystic pattern as MSA, but the targetoid pattern and perineural invasion of PAC are distinguishable features characterizing it from MSA. Regarding the immunoprofile, it is quite similar in both tumors. They are both positive for S100 and share the discrepant p63 positivity and p40 negativity [1, 3, 6], yet PAC shows positivity to mammaglobin to a great degree [1] (Fig. 7).

In the initial case series published by Bishop et al. to describe MSA, one of the control cases exhibited a related yet different fusion; *SS18-ZBTB7A* [7]. In 2021, Freiberger et al. identified a case of salivary adenocarcinoma harboring SS18-ZBTB7A [29]. Recently, in 2023 Weinreb et al. published a case series designating this tumor along with 3 other similar tumors sharing the same characteristic gene fusion as “microcribriform adenocarcinoma” (MCA) [30]. MCA shares similar features with MSA such as a cellular fibromyxoid stroma, bland cellular features, basophilic intraluminal secretions. Yet, there are some variations between them. Microcystic and tubular growth patterns as well as profuse intraluminal secretions are more evident in MSA, while solid, cribriform, and single cell patterns were more detected in MCA. Also, MCA has characteristic oncocytic cells in a glandular pattern. Regarding the immuno profile, both MSA and MCA are positive for S-100 and SOX10. However, MCA exhibit focal p63 and p40 positivity in the outer cells lining tubules and nests suggesting myoepithelial cell component and biphasic tumor growth [30]. These findings underpin the ever-evolving recategorization of SGTs previously diagnosed as NOS and emphasizes the importance of their re-evaluation using molecular diagnostic techniques.

Histopathological features distinguishing MSA and other SGTs are highlighted in Table 3.

**Table 3:** Comparing histological features of microsecretory adenocarcinoma with other salivary gland tumors

Points of differentiation	Microsecretory adenocarcinoma	Other salivary gland tumors				
Cell lineage	Monophasic	**Monophasic** Secretory carcinoma Polymorphous adenocarcinoma		**Biphasic** Adenoid cystic carcinoma Epithelial myoepithelial carcinoma Pleomorphic adenoma Sclerosing microcystic adenocarcinoma		
Cell shape	Flat	**Different shapes** Pleomorphic adenoma Myoepithelioma	**Basaloid** Adenoid cystic carcinoma	**Vacuolated** Secretory carcinoma Acinic cell carcinoma Secretory myoepithelial carcinoma		**Signet-ring** Mucinous adenocarcinoma Secretory carcinoma Secretory myoepithelial carcinoma
Growth pattern	Tubules Cords Cribriform	**Duct-like pattern** Pleomorphic adenoma Sclerosing microcystic adenocarcinoma	**Tubules** Pleomorphic adenoma Myoepithelioma Adenoid cystic carcinoma Sclerosing microcystic adenocarcinoma Polymorphous adenocarcinoma Secretory carcinoma	**Cribriform** Adenoid cystic carcinoma Epithelial myoepithelial carcinoma Polymorphous adenocarcinoma Microcribriform adenocarcinoma	**Papillary** Acinic cell carcinoma Mucinous adenocarcinoma Salivary duct carcinoma Polymorphous adenocarcinoma	**Single cell pattern** Polymorphous adenocarcinoma Micro cribriform adenocarcinoma
Cyst component	Microcystic	**Microcystic** Polymorphous adenocarcinoma Sclerosing microcystic adenocarcinoma Secretory carcinoma	**Macrocystic** Mucinous adenocarcinoma	**Variable** Mucoepidermoid carcinoma		
	Fibromyxoid	**Mucoid, Hyalinized** Polymorphous adenocarcinoma Adenoid cystic carcinoma	**Myxoid**,** hyalinized**,** chondroid**,** fibrous**, Pleomorphic adenoma Myoepithelioma	**Fibrous** Sclerosing microcystic adenocarcinoma		

Cutaneous MSA is like other adnexal tumors resembling their salivary counterparts and have similar histological features and gene fusions. Some adnexal tumors should be considered in the primitive diagnosis along with cutaneous MSA such as secretory carcinoma, tubular adenoma, cribriform carcinoma, chondroid syringoma and microcystic schwannoma. Compared to cutaneous MSA, secretory carcinoma shows eosinophilic secretions, plump and vacuolated cytoplasm, and less prominent fibromyxoid stroma. Tubular adenoma exhibits round, oval, or irregularly shaped tubules that have a consistent dual or multilayered epithelium. The lumens of tubular adenoma have rare secretions and are larger than those seen in MSA. Cribriform carcinoma consists of multiple interconnected solid aggregations of tumor cells with round or oval, hyperchromatic, slightly pleomorphic nuclei, and scant eosinophilic cytoplasm. The solid tumor aggregates enclose small round spaces producing a cribriform pattern. Cribriform pattern is not common in MSA [8, 31]. The discordant positivity for p63 and negativity for p40 is very helpful in differentiating MSA from cribriform carcinoma, secretory carcinoma and tubular adenoma. Chondroid syringoma presents as a well-circumscribed multilobulated tumor mass separated by fibrous septa. The stroma may exhibit a homogeneous bluish chondroid appearance but can also be myxoid. The tumor cells are cuboidal or polygonal forming nests or strands that give rise to ducts and tubules. IHC is not necessary for diagnosis of chondroid syringoma [32]. Microcystic schwannoma is characterized by a microcystic and reticular growth pattern, composed of anastomosing and intersecting spindle cells with vacuolated cells in a collagenous to myxoid stroma. Schwannoma is not positive for p63 [33].

### Genetic diagnosis and its implications

RNA-Seq followed by reverse transcription polymerase chain reaction (PCR) were carried out in most reported cases of MSA detecting *MEF2C::SS18* fusion. Moreover, 95.6% (22/23) of patients had *SS18* gene rearrangement detected by *SS18* break apart fluorescence in situ hybridization (FISH). 32/32 patients had *MEF2C::SS18* fusion detected by RNA-Seq (Table 4). The breakpoints of fusion were demonstrated in 97% (31/32) patients. They were identical in 27 patients: exon 7 of *MEF2C* gene and exon 4 of *SS18* gene. Four patients have break points at different locations: (2) exon 7 of *MEF2C* and exon 2 of *SS18* [9], (1) exon 5 of *MEF2C* and exon 4 of *SS18* [15], and (1) exon 10 of *MEF2C* and exon 5 of *SS18* [8].

**Table 4:** Molecular findings of microsecretory adenocarcinoma

Reference	Number of cases	SS18 FISH	RNA-Seq/PCR
Salivary cases			
Bishop et al., 2019 [7]	5	N/A	*MEF2C::SS18* (5/5)
Kawakami et al., 2020 [12]	1	N/A	*MEF2C::SS18*
Walsh et al., 2021 [14]	1	N/A	*MEF2C::SS18*
Bishop et al. 2021 [6]	24	13 /14	*MEF2C::SS18* (21/21)
Gui et al., 2022 [15]	1	+	*MEF2C::SS18*
Hosseini et al., 2022 [16]	1	N/A	N/A
Jurmeister P et al., 2022 [17]	1	+	*MEF2C::SS18*
Lu Y et al., 2024 [4]	1	+	N/A
Razack et al., 2024 [13]	1	+	*MEF2C::SS18*
**Cutaneous cases**			
Chan et al., 2022 [18]	1	+	N/A
Dibbern et al., 2023 [19]	1	+	N/A
Bishop et al., 2023 [8]	4	3/3	*MEF2C: SS18* (3/3)
Bogiatzi et al., 2023 [9]	3	N/A	*MEF2C: SS18* (3/3)
Novice et al., 2024 [20]	1	N/A	*MEF2C: SS18*

/ numerator is the number of positive cases, denominator is the total number of cases in which the assay was done

N/A not available

Six cases in [6] were reported previously in [7] and [12]

Both salivary and cutaneous MSAs harbour the same *MEF2C::SS18* fusion. *MEF2C* (Myocyte Enhancer Factor 2 C) gene is an oncogene implicated to drive hematologic cancers [34]. It is quite interesting to note that a novel *MEF2C::SS18L1* gene fusion has been recently discovered in a child with acute B-lymphoblastic leukemia (B-ALL) [35]. *MEF2D::SS18* fusion is also detected in acute lymphoblastic leukemia (ALL) [36]. Moreover, SS18L1 is a homolog for the *SS18* gene and both *SS18* and *SS18L1* are detected in patients with synovial sarcoma [37]. It is therefore credible to presume a resemblance between the *MEF2C::SS18* fusion in MSA and the *MEF2C::SS18L1* fusion in B-ALL. Moreover, p63 and S100 are characteristically positive in all cases of MSA and these two markers are also implicated in hematologic tumors [35]. These observations strengthen the incrimination of these fusions in malignancy. But the scope and full implications of this similarity is yet to be elucidated.

*MEF2C* is also suggested to have a pro-oncogenic function in other tumors. Examples include but are not limited to, colorectal cancer, Hodgkin’s lymphoma and rhabdomyosarcoma. *MEF2C* is associated with breast cancer invasiveness [38]. Recently, *MEF2C* is established as a contributor to breast cancer brain metastasis [39, 40]. One of the reported cases of cutaneous MSA had a history of breast cancer [19]. The case diagnosed in our department also had a family history of breast cancer affecting all her aunts, only her mother was spared. This may strongly imply a role for *MEF2C*, and a possible link between tumors that have an abnormal expression of the *MEF2C* gene.

NGS demonstrating *MEF2C::SS18* gene fusion is accurate yet not available in most laboratories around the world especially in developing countries. It also requires a significant amount of RNA that is not always available from archived cases or small biopsies. *SS18* break-apart FISH is more readily available as it is already widely used as an aid in the diagnosis of synovial sarcoma. It can also be performed on smaller specimens. Bishop et al. carried out *SS18* break-apart FISH on four known cases of MSA as well as on tissue microarrays of a large number of various other SGTs. They demonstrated that *SS18* break-apart FISH is as accurate, sensitive and 100% specific for MSA where all other 374 SGTs were negative for *SS18* rearrangement [11]. However, some tumors with positive *SS18* rearrangement may be actually MCA, the most recent SGT entity [30], with a different fusion partner *ZBTB7A* unlike MSA with fusion partner *MEF2C.* So, it is essential to give great attention to histologic features of each single case and not only rely on the molecular findings.

Having discussed the significance of molecular testing and genetic diagnosis, it is important to stress that diagnosis should primarily depend on histology looking for a focus of classical tumor component since molecular tests do not always reveal positive results [41]. Moreover, negative results of molecular tests cannot fully exclude certain diagnosis due to presence of more than one genetic abnormality in tumors other than the well-known ones [10].

### Treatment and prognosis

Treatment of MSA is achieved by surgery [6]. Adjuvant chemoradiation was done in one case. This case was presented with lymph node metastasis (Pathologic stage: pT4bN3b) [15]. Many cases have an adequate disease-free follow up period proposing the low-grade nature of MSA, however few cases showed aggressive histological findings such as solid growth pattern, nuclear pleomorphism, high mitotic rate, and necrosis [8, 15]. The clinical presentation of almost all reported cases is a slowly growing painless mass which is also in favor of low-grade malignancy. Follow up period was not available for some cases. A case reported by Jurmeister et al. developed distant lung metastasis after one year and local recurrence after seven years of the initial tumor diagnosis [17]. Four years later, the patient medical condition was stable with only minimal growth of the metastatic lung nodules that were resected [17]. The reported cases of MSA unequivocally prove the malignant, yet low grade nature of MSA and call for long term follow up of newly diagnosed cases.

## Conclusions

Molecular tests like FISH, RNA-Seq or PCR are helpful to detect genetic abnormality in challenging cases to ensure accurate diagnosis. It cannot be denied that molecular analysis has helped in categorizing SGTs, confirming diagnosis of difficult cases and introducing new entities into SGT classification. The combined histopathologic, immunohistochemical and molecular profile of MSA is consistently uniform along almost all documented cases. Accordingly, with the increasing number of diagnosed MSA and pathologists being more familiar with its histologic pattern, molecular tests may not be necessary in straightforward cases and initial diagnosis can be validated with a confirmatory immunophenotype.

## Data Availability

No datasets were generated or analysed during the current study.

## Abbreviations

SGTs: Salivary Gland Tumors
NOS: Not Otherwise Specified
RNA-Seq: RNA sequencing
MSA: Microsecretory Adenocarcinoma
IHC: Immunohistochemistry
AdCC: Adenoid Cystic Carcinoma
PAC: Polymorphous Adenocarcinoma
SC: Secretory Carcinoma
MEC: Mucoepidermoid Carcinoma
PA: Pleomorphic Adenoma
EMC: Epithelial Myoepithelial Carcinoma
SMC: Secretory Myoepithelial Carcinoma
SMA: Sclerosing Microcystic Adenocarcinoma
MCA: Microcribriform Adenocarcinoma
PCR: Polymerase chain reaction
FISH: Fluorescence in Situ Hybridization
B-ALL: Acute B-lymphoblastic Leukemia
ALL: Acute Lymphoblastic Leukemia
NGS: Next-Generation Sequencing
